# Ocean rogue waves and their phase space dynamics in the limit of a linear interference model

**DOI:** 10.1038/srep35207

**Published:** 2016-10-12

**Authors:** Simon Birkholz, Carsten Brée, Ivan Veselić, Ayhan Demircan, Günter Steinmeyer

**Affiliations:** 1 Max-Born-Institut, Max-Born-Straße 2A, 12489 Berlin, Germany; 2 Weierstraß-Institut, Mohrenstr. 39, 10117 Berlin, Germany; 3 Fakultät für Mathematik, Technische Universität Dortmund, Vogelpothsweg 87, 44221 Dortmund, Germany; 4 Institute for Quantum Optics, Leibniz Universität Hannover, Welfengarten 1, 30167 Hannover, Germany

## Abstract

We reanalyse the probability for formation of extreme waves using the simple model of linear interference of a finite number of elementary waves with fixed amplitude and random phase fluctuations. Under these model assumptions no rogue waves appear when less than 10 elementary waves interfere with each other. Above this threshold rogue wave formation becomes increasingly likely, with appearance frequencies that may even exceed long-term observations by an order of magnitude. For estimation of the effective number of interfering waves, we suggest the Grassberger-Procaccia dimensional analysis of individual time series. For the ocean system, it is further shown that the resulting phase space dimension may vary, such that the threshold for rogue wave formation is not always reached. Time series analysis as well as the appearance of particular focusing wind conditions may enable an effective forecast of such rogue-wave prone situations. In particular, extracting the dimension from ocean time series allows much more specific estimation of the rogue wave probability.

Ocean rogue waves are large and suddenly appearing surface gravity waves^1^, which may cause severe damage to ships and other maritime structures^2^,^3^,^4^,^5^. Statistically similar anomalies have been observed for a variety of physical systems. In particular, optical rogue waves have been observed in nonlinear fiber propagation^6^, in speckle formation^7^, and in multiple filamentation^8^. In all these different physical systems, these extreme waves appear more frequently than predicted by Gaussian statistics, and their appearance frequency is described by heavytail probability density functions. Despite years of research, their exact origin, in particular that of ocean rogue waves, is still a matter of debate^9^. Linear interference of waves with random phase is certainly the oldest and most straightforward explanation for these extreme waves^10^. For the limiting case of an infinite number of interfering waves on the ocean surface, i.e., the formation of short-crested wave patterns, a Rayleigh probability density function


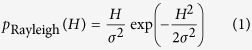


results, where *H* is the wave height (crest to trough) and *σ* a scale parameter. In the case of one-dimensional interference, in contrast, long crests form and the distribution is Gaussian. For the ocean system, careful comparisons with large records of wave height observations indicate, however, significant deviations from either model case^11^,^12^,^13^. An empirical function *p*_Forristall_(*H*) has been suggested that mediates between the short-crested and long-crested waves and provides a much better fit to long-term observations^12^,^14^.


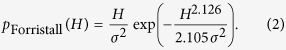


More recent explanations suggested that nonlinearities in the system could play a significant role^2^,^3^,^15^,^16^,^17^, in particular as inspired by the analogous case of nonlinear optical effects in fibers^6^,^8^. Other more advanced suggestions indicated breather solitons^18^ or the Peregrine soliton^19^ as the source of rogue waves. In support of a nonlinear origin of rogue waves, the soliton scenario has been confirmed in one-dimensional water channel experiments^20^. It nevertheless appears difficult to derive probability density functions from soliton theory. In the following, we show that nearly all aspects of ocean rogue waves can be explained by a linear model when the previous assumption of an infinite number of interfering waves is dropped. In particular, the linear interference model can explain observed probability density functions as well as the characteristic single-cycle nature of rogue wave observations. Nonlinearities are only required to explain the rare appearance of rogue holes.

## Conditional linear interference model

To this end, we assume linear interference of *N* elementary waves, giving rise to the surface elevation^21^


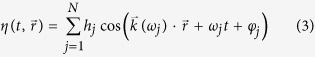


varying with time *t* and position 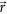. Here the wave amplitudes and phases are given by *h*_*j*_ and *φ*_*j*_, respectively. The wave vectors 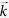
(*ω*_*j*_) are connected to the angular frequencies *ω*_*j*_ by the dispersion relation. The *ω*_*j*_ are incommensurable frequencies, which are distributed to provide a discrete representation of the wave spectrum. While the discrete nature of our model may appear as a limitation at first sight, this issue can be overcome by frequently recomputing the random seeds for the *ω*_*j*_, e.g., such that their average probability density function converges to the JONSWAP spectrum^22^. To leading order, the nonlinearity of the system can be accounted for by adding second harmonic contributions 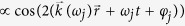
21. This correction lifts the crests as much as the troughs and therefore does not affect the crest-to-trough wave height. Higher-order corrections can be implemented as well, but have been estimated to lie in the centimeter range even for a rogue wave^23^. As the time series are sampled at one point in space, we set 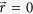, which makes us independent of the dispersion relation of the system. Sampling the surface elevation at a rate ≪ *ω*_*j*_ then gives access to the statistics of surface elevation and wave height by repeatedly computing


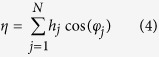


and


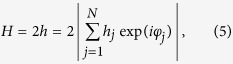


respectively, for randomly chosen amplitudes *h*_*j*_ and phases *φ*_*j*_. The problem of the resulting amplitude *h* of *N* interfering waves is mathematically equivalent to computing the probability of the length *h* of two-dimensional random walks^10^,^24^,^25^ involving *N* steps. In the limiting case of *N* → ∞, identical probability density functions emerge independent of the amplitude distribution. In the following, we therefore set *h*_*j*_ = const. for simplicity. Moreover, we only consider wave amplitudes and wave heights, as their statistics do not require any correction for ocean nonlinearities to leading order. This makes our model fairly generally applicable, e.g., for explaining speckle pattern formation in multimode fibers^7^, formation of caustics with a light modulator^26^, as well as extreme events in microwave experiments^27^. Moreover, in all of these experiments, linear interference appears in two or more dimensions, which is in line with the prediction of ref. 10 that one-dimensional linear interference does not suffice to explain a heavy tail in the probability density function. The situation of a small number of waves has been mathematically investigated^24^,^25^, yet no analytical solution is known as a general function of the parameter *N*.

**Figure 1:**
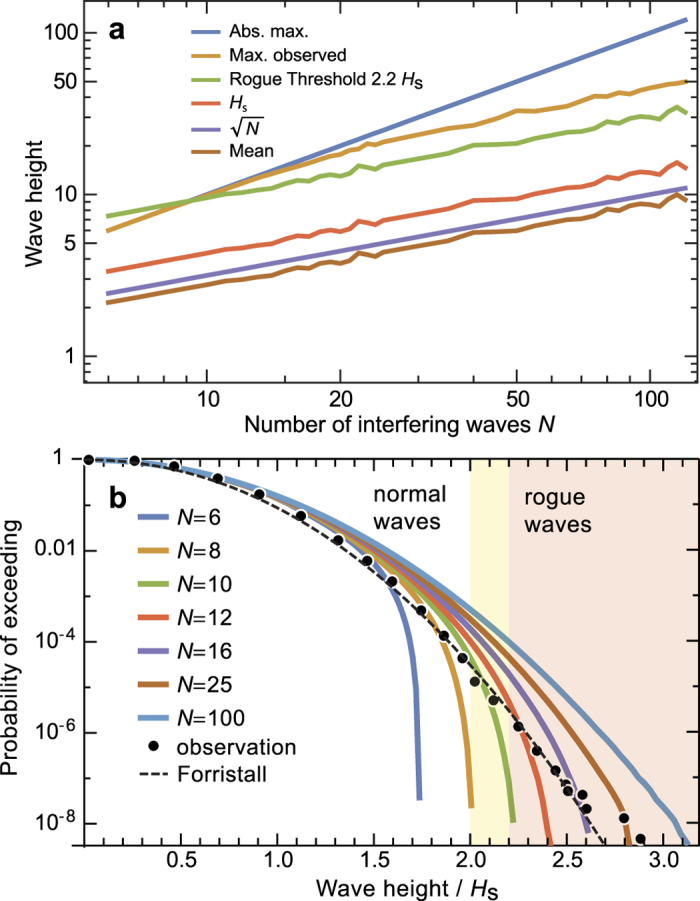
Simulation of statistical distributions resulting from the interference of *N* waves with unity amplitude and random phases. For each value of *N*, 10^9^ resulting wave heights (crest to trough) have been computed, i.e., about 4 times more than available in ref. 12. (**a**) Resulting average wave height, significant wave height *H*_S_ as well as maximum observed wave height as a function of *N*. For identical phases, a maximum possible wave height results at *N* times the height of the individual input waves. Using the threshold condition of 2.0 or 2.2*H*_S_, no rogue waves are possible below *N* = 8 or 10, respectively. (**b**) Computed probabilities of exceeding a given wave height in units of *H*_S_. Cases from *N* = 6 to 100 are shown as lines. Observations from Christou and Ewans are shown as dots^12^. The Forristall distribution is shown as a dashed line^14^. The fact that no single simulation fits to the observations is indicative of a strong variation of *N* due to changing weather conditions during the observations.

We numerically computed the resulting probability density functions for a finite number of interfering waves using Eq. (5) with univariate uncorrelated random phases *φ*_*k*_ ∈ [0, 2*π*], see Fig. 1. For the examples shown in Fig. 1, a total of 10^9^ random sets has been employed for any of the shown values of *N*. Assuming equal and constant wave amplitudes *h*_*j*_ = 1/2, the absolute maximum possible resulting crest-to-trough wave height *H* is *N*, and for sufficiently large *N* the mean height is 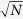, cf. Fig. 1(a). The threshold for rogue waves (2.2*H*_S_) scales as 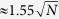. If all *φ*_*k*_ are identical, the maximum possible amplitude of *H*_max_ = *N* occurs. Comparing the rogue threshold with the maximum possible wave height, it becomes immediately clear that a minimum of 10 linearly interfering waves is required to exceed the rogue threshold. The resulting probability densities *p*_*N*_(*H*) are skewed, and a heavy tail starts to form with increasing *N*, see Fig. 1(b). For large (but finite) values of *N*, the probability function converges against the Rayleigh function *p*_R*ayleigh*_(*H*), as discussed in the literature^21^. We further tested a robustness of the probability density functions against the choice of an underlying wave spectrum, but could not detect any direct influence. However, we noticed that variations in the 1% range may occur when counting zero crossings rather than directly referring to the period of the central frequency. Nevertheless, these variations are too small to explain the systematic overestimation of the probability of rogue waves by the Rayleigh function.

## The Grassberger-Procaccia Algorithm

For a forecast of rogue waves, it would therefore be of high interest to estimate the number *N* of interfering waves, ideally using only a time series of surface elevation *η*(*t*) measured at one single location in the ocean. When amplitude variations can be neglected, *N* is identical to the dimension *D* of the phase space governing the dynamics of *η*(*t*). For small values of *D*, nonlinear time series analysis offers methods to estimate the phase space dimension. While originally proposed for reconstructing the attractor dimension in chaotic systems, the Grassberger-Procaccia analysis (GPA)^28^,^29^ is a proven tool for extraction of a dimension estimate *D*_c*orr*_ from one-dimensional time series 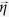 = {*η*_1_, *η*_2_, *η*_3_, …, *η*_*n*_} of surface elevations^30^,^31^ with record length *n*. From this time series 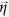, sub-series 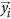 = {*η*_*i*_, *η*_*i*+1_, *η*_*i*+2_, …, *η*_*i*+*m*_} of length *m* are selected, with the embedding dimension *m*. Each sub-series 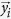 is then compared to all sub-series 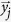 with *j* > *i* + m by calculating Euclidian distances


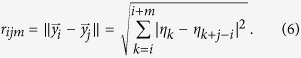


**Figure 2:**
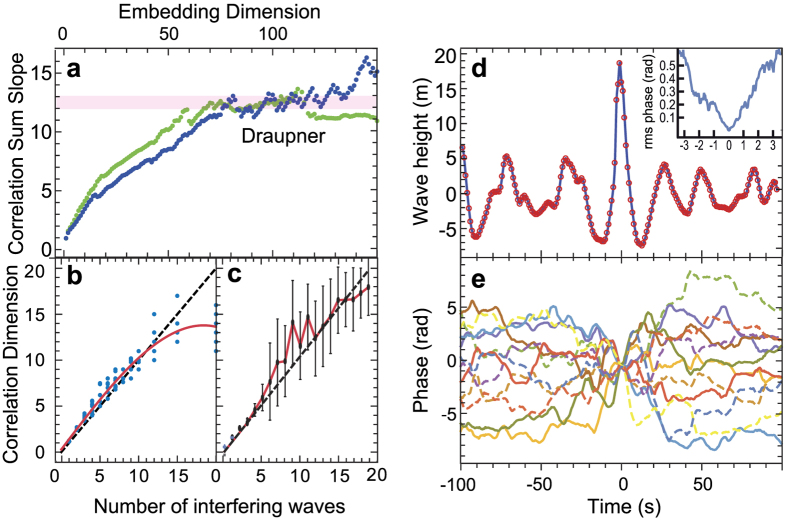
(**a**) Dimensional analysis of the Draupner wave record^32^ with the Grassberger-Procaccia method. Shown is the correlation sum slope as a function of embedding dimension for the original 15:20 Draupner record (green) and a second measurement from the same campaign starting at 16:20 (blue). The first record contains a rogue event. Both correlation sums exhibit a plateau of the slope for embedding dimensions ranging from 70 to 130. This results in an estimate for the information dimension *D* of the record of 12 to 13, see pink shaded area. (**b**) Reconstructed number of interfering waves *D* vs. actual number of interfering waves *N*. Analysis is based on 10 simulated time series according to Eq. (3) for each *N*. Sampling, duration, and the number of points have been chosen equal to the Draupner record. To leading order nonlinearity was corrected for. Points show the reconstructions, red line the resulting mean curve, and black line the ideal reconstruction. (**c**) Same with reconstruction by comparison with previously previously collected mean correlation sums. (**d**) Reconstruction of the Draupner event using the model assumption of an interference of 12 waves of equal and constant amplitudes but temporally varying phases *φ*_*j*_(*t*), (*j* = 1, …, 12), (original data: black curve, symbols: reconstruction). (**e**) Phase functions *φ*_*j*_(*t*) determined by simulated annealing. Around *t* = 0, linear approximations of *φ*_*j*_(*t*) fall into two separated groups. This separation is indicative of a crossing of two wave groups with well separated frequencies as was also discussed in ref. 23.

For *m* ≪ *n* the Euclidian distances *r*_*ijm*_ are accumulated in the correlation sum


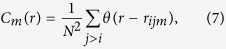


where *θ*(*r*) is the Heaviside step function. In order to avoid false interpretation due to detection noise and limited sample length, *C*_*m*_(*r*) is only analyzed at the interval [0.01 *r*_max_, 0.1 *r*_max_], where *r*_max_ is the largest Euclidian distance found in the data series. The correlation sum *C*_*m*_(*r*) increases monotonically according to


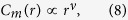


with the exponent *ν*. For small values of *m*, *ν* increases with embedding dimension *m* until it reaches a saturation value *d*_sat_. For a range of values of *m*, a plateau with nearly constant *D*_corr_ = *d*_sat_ is typically observed (cf. Fig. 2), provided a sufficient number of data points in the analysis.

## Validation of the GPA for Estimation of Phase Space Dimension

While the GPA has found frequent application in the analysis of chaotic data sets, its usefulness for retrieving the phase space dimension of the ocean dynamics from a time series of the surface elevation may appear debatable. We therefore generated a number of synthetic data sets based on our model Eq. (3). These data sets resemble the Draupner wave record^32^ concerning record length, number of sampled points, and covariance function. The randomly chosen frequencies *ω*_*i*_ in Eq. (3) have been chosen in accordance with the JONSWAP spectrum^22^, and the leading-order correction for the ocean nonlinearity has been applied. *N* has been varied from 1 to 20, and the data sets have then been processed with the GPA. Figure 2(b) shows the retrieved *D*_c*orr*_ as a function of *N*. This analysis indicates that one can quite reliably reconstruct the phase space dimension up to values of about 10. For larger values, a stagnation is observed. Nevertheless, retrieved values above 10 seem to safely show that the emergence of rogue waves is possible. Values significantly below 10 also quite clearly indicate the opposite. As clear identification of the plateau region is sometimes very difficult, we also tested a second way for reconstruction of the phase space dimension. To this end, we generated sets of 100 random-generated data sets for each value of *N*, which we then used to construct average correlation sums 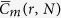. Subsequently, we produced further data sets at random *N* and then selected the average correlation sum that provided the best fit. The result of this test procedure is shown in Fig. 2(c). The phase space dimension can be nearly perfectly retrieved for *N* ≤ 6. For larger values, there is a sudden increase in the resulting standard deviation, but the reconstruction continues to follow *N* up to values of 16 before a stagnation also sets in for the second method. Considering that a duration of only 20 minutes of wave data was assumed here, a fairly reliable reconstruction of the phase space dimensions seems to be possible within about an hour of observation.

## Phase Space Dimension Estimates for Ocean Data

Previous studies directed at ocean dynamics during storms^30^,^31^ indicated values of *D*_corr_ reaching from 7 to 10.5, with a certain tendency of lower values appearing in calmer waters. Applying the same method to wave height measurements recorded on January 1, 1995 on the Draupner platform^32^, we determine a dimension *D*_corr_ in the range of 12 to 13, see Fig. 2(a). The underlying measurement [Fig. 2(d)] is the first record of a rogue wave, which ultimately confirmed their existence. The significant wave height *H*_S_ in this event was 12 m, and the rogue wave exhibited a wave height of *H* = 25.6 m (crest to trough). For comparison, we also ran the GPA on other data sets^8^ recorded during the Draupner storm, which provided a nearly identical result for *D*_corr_.

It is important to understand that the dimension is an estimate of the sea state, which can neither be obtained by linear statistical methods nor extracted from spectrum and autoconvolution function^33^,^34^,^35^. Moreover, the dimension is independent from the appearance of an actual rogue wave in the time series as one can see from comparison of the two traces in Fig. 2(a). Nevertheless, *D* indicates whether a rogue could have formed in a given sea state as well as the probability for its formation.

While one certainly has to be careful in interpreting these results, the comparison between the three different data sets appears to be consistent with expectations. Measurements at a fairly protected location off Venice^31^ lead to the lowest values of *D*_c*orr*_, whereas the extreme storm conditions in the North Sea at the Draupner platform are indicative of values nearly double as high. Moreover, a phase space dimension of 12 or higher clearly enables the emergence of rogue waves whereas the much lower values of *D* in previous measurements would not allow for the formation of extreme waves. We further ran a surrogate analysis^8^ on the Draupner data set, which resulted in an absence of the plateau region in Fig. 2(a), i.e., an undefined or infinite value of *D*. This systematic difference further supports our hypothesis of a finite number of interfering waves. One may further conclude that the complexity of the ocean dynamics is variable and depends both on location and time.

## Phase Diffusion and Rogue Waves

If the phase space dimension behind the ocean dynamics varies as suggested, then one probably observe relatively calm conditions during most of the time. These conditions simply do not allow for the formation of rogue waves, i.e., *D* < 10. Nevertheless, in order to explain the extensive statistics of wave records by Christou and Ewans^12^, *D* must occasionally reach very large values much larger than 12. A second explanation for the largest observed rogue events *H* > 2.5*H*_S_ in the Christou record may be nonlinear steepening or compression effects, which further amplify the heavy tail, similar as nonlinear corrections to linear statistics. The resulting waves have been previously discussed as super rogue waves^20^.

**Figure 3:**
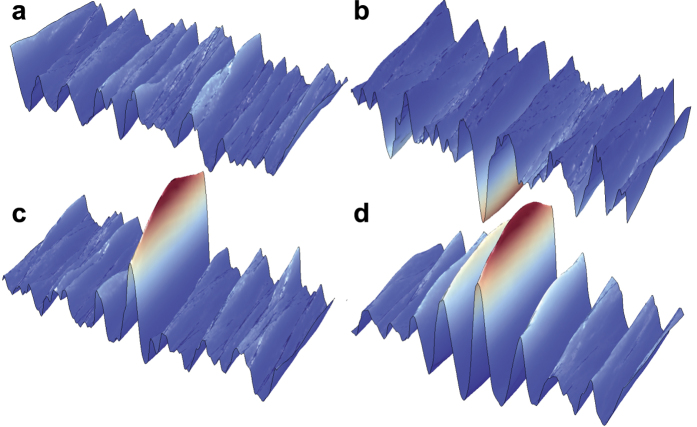
Simulation of the interference on a surface with a normally distributed angular spread of *N* waves amounting to 50 mrad. In this simulations, a nonlinear correction was included to reduce the depth of the troughs and steepen the wave crests. (**a**) Assuming random phases between the waves. A highly random wave pattern emerges with no wave significantly exceeding the significant wave height. *N* = 20. (**b**) Simulation of a rogue hole^37^, with synchronized phases adding up to negative interference at the central point of the propagation axis. *N* = 40. (**c**) Same with phase flipped by *π* and *N* = 20, resulting in a single wave with extreme height >2.5*H*_S_ Depending on the diffusion coefficient, leading and trailing troughs appear, similar to the observation of the Draupner event^32^. (**d**) Same with weaker phase diffusion. A rogue wave with structure reminiscent of the “three sisters”^38^ emerges.

Let us further verify the consequences of our model on *N* linearly interfering elementary waves. Assuming unity amplitudes, we can rewrite the resulting surface elevation


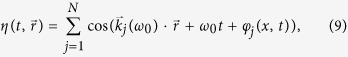


which assumes a distribution of angular frequencies around a central value *ω*_0_. The distribution of frequencies of Eq. (3) has now been carried into individual time and propagation dependent phases, reverting it to a phase diffusion problem. The wave vectors are defined as 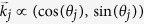, with a Gaussian distribution of random angles *θ*_*j*_ of vanishing mean. The standard deviation Δ*θ* of the angular distribution of waves determines the lateral extent of a rogue wave along the *y* direction. Using a 2*π* univariate angular distribution instead, one can simulate the emergence of circular symmetric rogue waves as observed by Arecchi *et al*.^7^. A one-dimensional diffusion process of the phase functions *φ*_*j*_(*x*, *t*) along the propagation direction^36^ is computed at equidistant positions *x*_*i*_ = *i*Δ_*x*_ according to the recursive law


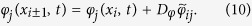


with a diffusion coefficient *D*_*φ*_ and a set of independent Gaussian random phases 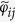 with mean zero. This model assumption induces a random phase walk, which nevertheless leaves the average frequency *ω*_0_ unaffected. In a nearly monochromatic case, no special assumptions for the dispersion of the system are required, but can certainly be included for more broadband scenarios. An example for the resulting random surface is shown in Fig. 3(a). Rogue waves appear by setting *φ*_*j*_ = 0 at *t* = 0 and *x* = 0, see Fig. 3(c,d); rogue holes^37^ can be generated by forcing all *φ*_*j*_ = *π* [Fig. 3(b)]. In the examples, the angular spread of the *θ*_*j*_ has been adjusted to 50 mrad to allow a certain vertical extension of the rogue waves. Narrowing down the spread, wider and wider walls of water appear. *N* has been set to 20, which exceeds the estimate from the dimensional analysis (*D* = 12 to 13). The characteristic length or time over which dephasing appears is related to the linear correlation time or the prediction length^8^. Decreasing the parameter *D*_*φ*_, one observes a transition from completely isolated rogue waves to the formation of rogue wave groups that resemble the famous “three sisters”^38^. The Draupner waveform with its characteristic leading and trailing deep troughs appears for intermediate values of *D*_*φ*_.

This very simple model can also be inverted to find a set of phase functions *φ*_*j*_(*t*) that give rise to a measured wave record *η*(*t*), cf. Fig. 2(d,e). Starting at the time *t* = 0 of the rogue event, we used a simple simulated annealing variant to adjust the individual phases *φ*_*j*_(*t*) in a round-robin fashion to the end of obtaining best agreement with the measured record of the Draupner event. This procedure is repeated for each time step in the measured time series. As this phase retrieval is an ill-posed problem, rapid temporal oscillations of the phase functions may locally appear. These oscillations can be mostly suppressed by a suitable penalty term, with the noted exception of the deep trough areas of the Draupner wave. This may be an indication for unaccounted nonlinear shaping in the immediate vicinity of the rogue wave. It should also be noted that phase retrieval is certainly highly ambiguous in this situation, but nevertheless indicates that rogue wave formation can be explained due to phase diffusion in the interference of a finite number of elementary wave with constant and equal amplitude. The phase dynamics near *t* = 0 shows essentially a linear dephasing behavior with time, apparently simply being caused by different frequencies of two groups of interfering waves. This reproduces the results of a previous analysis^23^, which attributed the Draupner wave to the interference of two crossing wave groups with markedly different frequencies. We have further investigated the influence of the wave spectrum on the resulting rogue wave. Using the JONSWAP spectrum together with 12 randomly chosen frequencies, we can reproduce rogue waves with shape similar to the Draupner wave, see supplementary movie. From this simulation, one can estimate a lifetime of the Draupner rogue wave of about 2 minutes. At *N* > 12 the lifetime of a rogue wave can be substantially longer. Narrowing down the spectral extension of the wave spectrum, we observe rogue wave groups similar to the “three sisters”^38^. The slower phase diffusion process for this type of rogue waves also leads to an increase of their lifetime.

In the completely linear picture of Eq. (3), one would expect to see rogue holes with the same probability as rogue waves. The leading-order nonlinearity correction, however, causes an equal uplift, both of the troughs and the crests. For the Draupner wave, this correction amounts to about 4 meters^23^, which would have rendered the emergence of an isolated rogue hole impossible under Draupner storm conditions. Apart from the rogue hole anomaly, however, rogue wave formation appears perfectly explainable by random linear interference of a limited number of elementary waves.

## Discussion

Our model relates the emergence of ocean rogue waves essentially only to the single parameter *N*, with the shape of the rogue wave being related to the wave spectrum. As we showed above, the effective number *N* of interfering waves can be estimated via the GPA analysis. The variable character of *N* therefore appears key to understanding the rogue wave phenomenon. As the waves are generated by winds across the ocean surface, one expects to see an increase of the average number of interfering waves, e.g., when wind directions change over a wide range or when turbulence sets in. The fact that rogue wave appearance is not simply correlated to the wave height, but also depends on the weather conditions was already suggested by Waseda *et al*.^13^. In particular, the latter authors showed that there is a correlation between rogue wave appearance in the North Sea and the presence of a pronounced low pressure region off the North Norwegian coast paired with a high-pressure cell in the Bay of Biscay. A similar metereological situation appeared on January 1, 1995, i.e., the date of the Draupner storm^32^. Here it appears striking that an auxiliary low with extremely narrow and strongly curved isobars must have crossed the position of the Draupner platform. This situation must have given rise to a strong focusing action of the winds and subsequently generated waves, in addition to a high directional variability of the winds. This situation is expected to give rise to crossing seas, i.e., when waves of different directions cross each other in an ocean region^39^. Let us further remark that the number of interfering waves may certainly also be influenced by particular coastal shapes, subsea topography, or ocean currents, which may have a focusing or cumulating effect on otherwise spatially separated waves^40^,^41^. All these effects may contribute to highly localized rogue wave formation, whereas no rogue waves appear in neighboring ocean regions with equally high waves. In fact, the strongest winds during the Draupner storm passed the platform several 100 km further to the west^32^. While all these indications further support our hypotheses of a linear origin of ocean rogue waves, there is certainly more work necessary to deduce effective rogue wave warnings from ocean weather reports.

As an alternative to meteorological prediction, we suggest use of the correlation dimension *D* for characterization of the sea state. As indicated by the example analysis of the Draupner data, relatively short time series of 20 minutes length already suffice for a coarse estimate of *D*, and required computational times for its determination are on the order of a second with current computer technology. Values above the threshold of *D* ≈ 10 indicate the possibility of rogue waves, with a rapidly increasing probability at higher *D*. Using time series analysis, it may therefore very well be possible to build “a device on the mast of a ship analyzing the surface of the sea”^42^ in order to obtain effective rogue wave warnings. To this end, we suggest application of the GPA to larger wave records^12^,^13^ for solidification of the suspected correlation between rogue wave appearance and large values of *D*_corr _. Finally, a further possibility for identifying rogue wave situations may be given by measuring the transverse extent of the wave crests. In the light of the above discussion, short-crested waves appear much more dangerous than long-crested ones, with the interference of a much wider angular spread^39^ of elementary waves in the two-dimensional case.

## Conclusions

In conclusion, our simple linear model indicates that ocean rogue waves may appear due to interference of elementary waves, similar to what was suggested by Longuet-Higgins^10^ already some 60 years ago, yet with a finite number of interfering waves. Nonlinearities may play a modifying role when present, but their presence is not strictly required to explain rogue wave formation. In particular, there remains the anomalously low appearance frequency of rogue holes, which cannot be explained without including nonlinear corrections. Minor modification of the original Longuet-Higgins theory, i.e., reducing it to a finite number of interfering waves, gives rise to a threshold condition of a minimum number of interfering waves that allow for rogue wave formation. This threshold condition may prove useful for an effective forecast of rogue waves in the ocean. We indicated possible ways to predict rogue-wave prone situations in the ocean, including a meteorological approach, observation of the wave crest extension, and time series analysis. One explanation for the observed variability of the dimension may be the changing wind-forcing of the waves, which is behind a majority of ocean waves. Compared to the role of nonlinearity, the aspect of wind-forcing has not found much attention in the rogue-wave literature so far. Nevertheless, further research is required to clearly identify wind conditions that give rise to dimensions that exceed the threshold condition for rogue wave formation.

## Additional Information

**How to cite this article**: Birkholz, S. *et al*. Ocean rogue waves and their phase space dynamics in the limit of a linear interference model. *Sci. Rep*. **6**, 35207; doi: 10.1038/srep35207 (2016).

## Supplementary Material

Supplementary Information

Supplementary Movie S1
