# Supplementary material for: Ocean rogue waves and their phase space dynamics in the limit of a linear interference model
Source: Sci Rep. 2016 Oct 12;6:35207. doi: 10.1038/srep35207 (PMC5059714; doi:10.1038/srep35207)
Supplement: Supplementary Information [file srep35207-s1.doc]

**Ocean rogue waves and their phase space dynamics in the limit of a linear interference model**

Simon Birkholz, Carsten Brée, Ivan Veselic, Ayhan Demircan, and Günter Steinmeyer

**Caption supplementary video material**

Numerical simulation of a Draupner-like rogue wave occurring due to linear interference of 12 waves of identical amplitude. The frequencies of the waves have been selected randomly according to a JONSWAP probability density. Phase and group velocity of the wave have been adapted to the storm conditions on January 1, 1995 in the North Sea. A correction for ocean nonlinearity has been included to leading order, lifting the crest and the troughs of the rogue wave equally by about 4 meters. These assumptions enable an estimation of lifetime and propagation distance of a rogue wave before falling below the threshold criterion (red line).
